# Characterization of the complete chloroplast genome of the *Helleborus atrorubens* Waldst. & Kit. (Ranunculaceae)

**DOI:** 10.1080/23802359.2022.2119105

**Published:** 2022-09-07

**Authors:** Xiaohua Shi, Lihui Mao, Liang Jin, Guangying Ma

**Affiliations:** Zhejiang Academy of Agricultural Sciences, Zhejiang Institute of Landscape Plants and Flowers, Hangzhou, China

**Keywords:** *Helleborus*, plastome, genome structure, phylogenomics

## Abstract

*Helleborus atrorubens* is an economically important perennial garden plant with medicinal value. Here, we sequenced the complete chloroplast genome of *H. atrorubens*. The results revealed the chloroplast genome to be 166,695 bp in length. It possesses a typical quadripartite structure containing one large single copy (LSC) region (84994 bp), one small single copy (SSC) region (17,825 bp), and a pair of inverted repeat (IR) regions (31938 bp). This chloroplast genome encoded 130 genes, out of which 85 code for proteins, 37 for transfer RNAs, and 8 for ribosomal RNAs. Simple sequence repeat (SSR) markers and the top variable coding regions were identified.

Our study lays a foundation for further research, such as species differentiation and phylogenetic reconstruction of the *Helleborus* genus.

*Helleborus atrorubens* is a perennial species native to the Balkan Peninsula with ornamental and medicinal value. While the diversity of phytochemicals produced by *H. atrorubens* has been well investigated, there is a dearth of genomic resources for this species (Male and Medic-Saric 2001). The genetic relationships in *Helleborus* are complex although there are only approximately 20 species in this genus. The English botanist Brian Mathew classified the genus into six groups, species within same group can hybridize with each other while species from different groups are hard to hybridize (Rice and Strangman 1993). It is necessary to find SSR and potential polymorphic gene fragments from the chloroplast genome to better analyze the genetic relationships among the species and varieties in *Helleborus*. *Helleborus atrorubens* were purchased from Het Wilgenbroeks farm (Oostkamp, Belgium. 51.17° N, 3.23° E). The plants were cultivated in Wangcun Village, Linpu Town, Xiaoshan District, Hangzhou, China (30.069° N, 120.232° E). A voucher specimen was deposited in the Herbarium of Northwest A&F University (NWFC) under voucher number Shi001 (contact person: Linjuan Du, dulingjuan@nwafu.edu.cn). The genomic DNA was extracted with a plant genomic DNA kit (Tiangen Biotech, Beijing, China) and sequenced using the Illumina NovaSeq platform according to the manufacturer’s recommendations.

Pair-end Illumina raw reads were cleaned from adaptors and barcodes and then quality filtered using Trimmomatic (Bolger et al. 2014). After the quality filter, reads were mapped to the plastome of *H. thibetanus*, the closest species with an available plastome, using Bowtie2 v.2.2.6 (Langmead et al. 2009) to exclude reads of nuclear and mitochondrial origins. Next, all putative plastid reads mapped to the reference sequence above were used for *de novo* assembly to reconstruct the plastome using GetOrganelle 1.7.5 (Jin et al. 2020). The clean reads were then mapped to the complete plastome again to examine and correct mis-assemblies. Automatic annotation of the plastome was generated by CpGAVAS2 (Shi et al. 2019) and then manually corrected according to previously published plastomes using Geneious V8.1 (Kearse et al. 2012). A circular representation of both sequences was plotted using the online tool OGDRAW (Greiner et al. 2019).

The complete plastome spans 166,695 bp and contains 130 genes, including 85 protein-coding genes, 37 tRNA genes, and 8 rRNA genes. A maximum-likelihood analysis was performed by RAxML v8.2.10 (Stamatakis 2014) using the GTR model and 1000 bootstrap replicates to confirm the phylogenetic position of *H. atrorubens*. Twenty-seven species in Ranunculaceae family were used in the phylogenetic analysis, including *Anemone reflexa*, *Anemoclema glaucifolium*, *Clematis terniflora*, *Oxygraphis glacialis*, *Ranunculus macranthus*, and another 21 species. The analysis revealed *H. atrorubens* to have the closest relationship with *H. thibetanus* (Figure 1). The result indicates that the phylogenetic analysis of Ranunculaceae based on the chloroplast genome is credible.

In this study, 106 chloroplast SSRs (cpSSRs) were identified for *H. atrorubens*, ranging in length from 10 to 18 bp. These SSR loci could be used to investigate the genetic diversity and genetic structure of natural populations and cultivars of this species. The nucleotide diversity (Pi) values were calculated using each gene to determine the hotspots of divergence. A total of 25 genes including *psbA, psbD, psbC* and others with a high Pi value (>0.01) and suitable length (>500 and <1500 bp) for PCR amplification can be used as candidate molecular markers to study interspecific relationships (Table S1).

**Figure 1.**
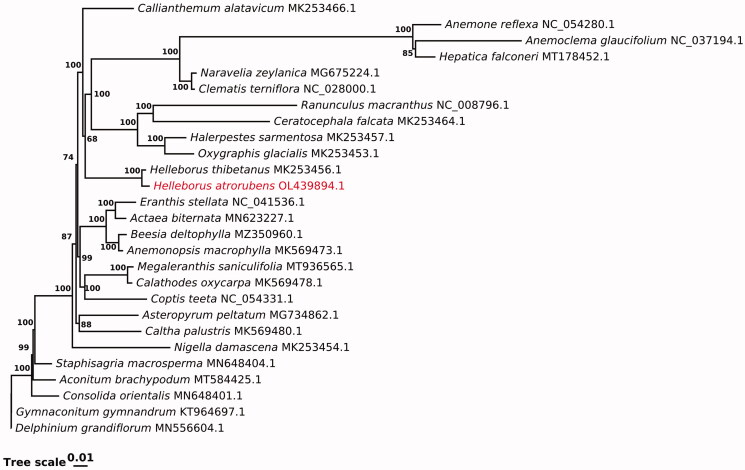
Molecular phylogenetic reconstruction of *Helleborus atrorubens* and 26 other Ranunculaceae species using maximum likelihood. Bootstrap support values (*N* = 1000) are indicated at each node.

In this study, we elucidated the complete plastome of *H. atrorubens* for the first time. The gene order and cp genome arrangement of *H. atrorubens* were similar to those of related species. The current study provides a valuable contribution that could help species identification, crossbreeding and genetic diversity research.

## Data Availability

The data that support the findings of this study are openly available in GenBank of NCBI at https://www.ncbi.nlm.nih.gov, reference number OL439894. The associated **BioProject**, **SRA**, and **Bio-Sample** numbers are SAMN26535971, SRS12222224, and *Helleborus atrorubens* respectively.
